# Genome-wide investigation and expression profiles of the *NPF* gene family provide insight into the abiotic stress resistance of *Gossypium hirsutum*

**DOI:** 10.3389/fpls.2023.1103340

**Published:** 2023-01-19

**Authors:** Juanjuan Liu, Caixiang Wang, Jialuo Peng, Jisheng Ju, Ying Li, Chaozhou Li, Junji Su

**Affiliations:** State Key Laboratory of Aridland Crop Science, College of Life Science and Technology, Gansu Agricultural University, Lanzhou, China

**Keywords:** cotton, *NPF* genes, genome-wide identiﬁcation, abiotic stresses, gene expression

## Abstract

Membrane transporters encoded by *NITRATE TRANSPORTER 1/PEPTIDE TRANSPORTER* (*NPF*) genes, which play crucial roles in plant growth, development and resistance to various stresses, are involved in the transport of nitrate (NO_3_^-^) and peptides. In several plant species, *NPF* genes are involved in the resistance to abiotic stresses; however, whether the whole *NPF* gene family in cotton contributes to this resistance has not been systematically investigated. Here, 201 genes encoding *NPF* proteins with a peptide transporter (PTR) domain were confirmed in three different *Gossypium* species, namely, *Gossypium hirsutum*, *Gossypium arboreum* and *Gossypium raimondii*. The NPF proteins in these three *Gossypium* species and *Arabidopsis thaliana* were classified into three different subfamilies *via* phylogenetic analysis. Among the genes that encode these proteins, most *GhNPF* genes in the same subfamily contained similar gene structures and conserved domains. Predictions of the promoters of these genes revealed that the cis-acting elements included phytohormone- and light-responsive elements, indicating that some of these genes might be expressed in response to abiotic stress. Furthermore, 52 common potential candidate genes in 98 *GhNPFs* were predicted to exhibit specific spatiotemporal expression patterns in different tissues based on two RNA sequencing (RNA-seq) datasets. Finally, the gene expression profiles of abiotic stress indicated that 31 *GhNPF* genes were upregulated in at least one treatment period. Under abiotic stress for 12 and 24 h, the expression of *GhNPF8* was upregulated upon cold treatment but downregulated with heat treatment, salt treatment and drought treatment. Furthermore, the expression of genes *GhNPF8, GhNPF54* and *GhNPF43* peaked at 6 h after heat and salt treatment. These results indicated that these genes exhibit underlying characteristics related to responses to abiotic stress. The verification of *NPFs* and analysis of their expression profiles in different tissues and in response to different abiotic stresses of cotton provide a basis for further studying the relationship between abiotic stress resistance and nitrogen (N) transport in cotton, as well as identifying candidate genes to facilitate their functional identification.

## Introduction

Abiotic stressors, such as heat, cold, drought, and salinity, are major threats and can markedly reduce plant quality and productivity (Deinlein et al.,2014; Drechsler et al., 2018; Hossain et al., 2018). In response to these extremely adverse conditions, plants have developed comprehensive signaling systems to counteract and avoid adverse effects of environmental stress (Saeed et al., 2012). Stress sensing and signal transduction, which initiate a transduction cascade likely comprising multiple components, are important parts of plant response mechanisms. Studies have shown that the signaling functions of reactive oxygen species (ROS), reactive nitrogen species (RNS) and reactive carbonyl species (RCS) regulate plant resistance to abiotic stresses by regulating gene expression and protein posttranslational modification (Hossain et al., 2018), such as those of *OsAPX2* in *Oryza sativa* (Chou et al., 2012) and *LeNHX3* in *Lycopersicon esculentum* (Villalta et al., 2008). Additionally, climate change also directly and indirectly affects plant nutrition. Research has shown that when the concentration of CO_2_ increases, the nitrogen (N) content of plants decreases (Taub and Wang, 2008). Therefore, the pivotal regulatory factors of the nutrient signaling pathway also have a crucial effect on plants (Gong et al., 2020). It has been reported that the phosphate starvation response (PSR) is enhanced by directly enhancing the activity of the phosphate starvation response (PHR) gene in *Arabidopsis thaliana* (Rubio et al., 2001; Bustos et al., 2010) and that N use efficiency can be improved by *NRT1.1B* transport in rice (Zhang et al., 2019). These results indicated that *NRT1* can improve N use efficiency and thus can improve plant quality and productivity under adverse conditions.

*NRT1/PTR*, which is also named nitrate transporter 1/peptide transporter (*NPF*), is a type of low-affinity transport system (LATS) of N or NO_3_^-^ (Fan et al., 2017). The *NPF* gene family is the most abundant subfamily that encode NO_3_^-^ transporters in plants (O’Brien et al., 2016). The earliest cloned plant nitrate (NO_3_^-^) transporter gene was *NRT1.1* (also known as *NPF6.3* or *CHL1*) in *Arabidopsis*, which had been involved in both low- and high-affinity NO_3_^-^ transport (Ho et al., 2009; Wang et al., 2018). The absorption of NO_3_^–^ and ammonium-N in plants involves a major process mediated by NO_3_^–^ and ammonium-N transporters, respectively. Assimilation of N includes the reduction of NO_3_^-^ to ammonium, which eventually is incorporated into amino acids (aa) through an assimilation process (Goel and Singh, 2015). In plants, a number of processes, including N absorption and assimilation, are negatively influenced by extreme temperature, salt and drought (Goel and Singh, 2015). NO_3_^-^ is redistributed in plants under stress conditions, and this phenomenon occurs partly in response to the decreased expression of *NRT1.1* and *NRT1.5* (Zhang et al., 2014; Goel and Singh, 2015; Taochy et al., 2015). There is evidence that different stresses cause NO_3_^-^ assimilation by redistribution, which is transmitted by NO_3_^–^transport proteins NRT1.5 and NRT1.8 (Zhang et al., 2014). The expression levels of *NRT1.1* and *NRT1.5* in *Brassica juncea* and *Arabidopsis* are downregulated in response to 24 h of salt and drought stresses (Goel and Singh, 2015; Taochy et al., 2015), and the expression of *PtrNPF2.1* and *PtrNPF7.4* in *Poncirus trifoliata* is also induced by salt stress (Zhao et al., 2022). Research has shown that the supply of exogenous N to sorghum and tomato can efficiently moderate Na^+^ uptake and increase the K^+^ content in plants (Miranda et al., 2016; Singh et al., 2016). Exogenous N can also alleviate the uptake of Cl^-^ and Na^+^ in mustard under salinity stress (Jahan et al., 2020). In wheat, drought stress limits N translocation during the grain filling period, resulting in decreased yields (Kirda et al., 2001). In addition, high temperature can also inhibit N absorption and assimilation in wheat, rice and creeping bentgrass (Tahir and Nakata, 2005; Rachmilevitch et al., 2006; Ito et al., 2009; Ercoli et al., 2010), and the expression of *BJNRT1.1* is downregulated after 24 h of hot and cold treatment in *B. juncea* (Goel and Singh, 2015). Taken together, the results of these studies indicated that the *NPF* genes that are related to N transport may have a potential effect on the growth and development of plants under abiotic stress.

Cotton (*Gossypium* spp.) is an economically essential crop species in China, and cotton growth and development are intimately tied to water and fertilizer. Moreover, cotton is very sensitive to N (Zheng et al., 2018). Studies have shown that N fertilizer can improve cotton yield and contribute to drought stress tolerance through increased N metabolism (Zhang et al., 2019; Iqbal et al., 2020). Under conditions of salt stress, fertilization can improve the salt resistance of cotton and can substantially increase cotton yields (Dai et al., 2013). These findings suggest that plant growth and productivity under stress conditions can be best achieved by improving N use efficiency. In addition, the *GhNPF6.14* gene affects growth and nitrogen uptake and accumulation of cotton (Dong et al., 2022). Nevertheless, the *NPF* gene family has been poorly characterized in abiotic stress response of cotton. In this study, by performing a whole-genome analysis, we comprehensively identified 201 *Gossypium NPF* genes (including those in *Gossypium arboreum*, *Gossypium raimondii* and *Gossypium hirsutum*). Then, chromosome distributions, collinearity, motifs, gene structures, cis-acting element compositions and phylogenetic relationships were investigated. Additionally, the expression patterns of 98 *GhNPFs* in different tissues and under different abiotic stresses were systematically analyzed by RNA sequencing (RNA-seq) performed by staff at Zhejiang University and the Cotton Research Institute of CAAS (CRI) and by quantitative real-time PCR (qRT−PCR) techniques. The results provide a theoretical foundation for further elucidating the role and molecular mechanism of *GhNPF* genes in the abiotic stress response of cotton.

## Materials and methods

### Identification and prediction of amino acid characteristics of *NPF* gene family members in cotton

The amino acid sequences of *Arabidopsis NPFs* were used as references. The hidden Markov model (HMM) model file (PF00854) for the *AtNPF* gene was obtained from the Pfam database (El-Gebali et al., 2019). Then, HMME 3.0 software (Finn et al., 2015) was used to search for homologous genes in three *Gossypium* species (Zhu et al., 2017), with an *E-value* < 1e^-5^, and the preliminary candidate genes were identified after we omitted incorrect and redundant members. Finally, SMART (https://smart.embl.de/smart/set_mode.cgi?NORMAL=1), PfamScan (https://www.ebi.ac.uk/Tools/pfa/pfamscan/) and the NCBI Conserved Domain Database (CDD) (https://www.ncbi.nlm.nih.gov/cdd) online websites were used to further confirm whether these candidate NPF proteins contained conserved domains. The physicochemical properties of the GhNPF proteins were predicted using the online website ExPASy (https://web.expasy.org/compute_pi/). The subcellular localizations of GhNPF proteins were predicted using the WoLF PSORT online website (https://wolfpsort.hgc.jp/). Genomic datum for *Arabidopsis*, *G. hirsutum*, *G. raimondii* and *G. arboreum* were obtained from The Arabidopsis Information Resource (TAIR) (https://www.arabidopsis.org/index.jsp) and Cotton Functional Genomics Database (CottonFGD) (https://cottonfgd.net/about/download.html), respectively. TBtools 1.098745 (Chen et al., 2020) software was used to map the locations of the genes on the chromosomes, and genes were named according to the chromosomal locations of the *NPF* gene family in *G. hirsutum* species.

### Multiple sequence alignment and phylogenetic analysis of the *NPF* gene family

To identify tandem and segmental duplication events of *NPF* genes, a multiple sequence alignment of full-length NPF proteins was performed by MCScanX; for this, whole-genome sequences of *G. hirsutum*, *G. arboreum* and *G. raimondii* and gene annotations were used. Plots of the data were created using TBtools (Chen et al., 2020) software. To assess the evolutionary constrictions on each gene pair, the non-synonymous (Ka) and synonymous (Ks) substitutions were calculated using the Simple Ka/Ks Calculator (NG) in TBtools (Chen et al., 2020). To further observe the interspecific and intraspecific homology of the *NPF* genes, phylogenetic trees were constructed based on the NPF protein sequences of *Arabidopsis*, *G. hirsutum*, *G. arboreum* and *G. raimondii*. The ClustalW tool of MEGA-X software (Kumar et al., 2018) was used to align the protein sequences of the cotton and *Arabidopsis NPF* gene family members, and then the neighbor-joining (NJ) method was used to construct a phylogenetic tree; the Poisson model was used, and the bootstrap value was 1,000. Finally, the online tool iTOL (https://itol.embl.de/upload.cgi) was used to produce a high-quality phylogenetic tree map.

### Structure and conserved motif analysis of the *GhNPF* genes

The structures of the *GhNPF* genes were investigated on the basis of the *G. hirsutum* genome annotation data *via* the Visualize Gene Structure tool in TBtools (Chen et al., 2020). The conserved motifs of the *GhNPF* genes were explored *via* the online website MEME (https://meme-suite.org/meme/doc/meme.html), and the maximum base numbers were set to 10, with the default parameters used. The Gene Structure View tool in TBtools (Chen et al., 2020) was used to illustrate the gene structures and construct conserved motifs maps.

### Analysis of *cis*-acting elements in the promoters and gene ontology of *GhNPF* gene family members

To understand the possible regulatory and response mechanisms of *GhNPF* genes, the promoter region was selected for analysis. For this purpose, the 2,000 bp nucleotide sequence upstream of the start codon of the *GhNPF* family members were obtained from the CottonFGD (https://cottonfgd.net/about/download.html). The online website PlantCARE (http://bioinformatics.psb.ugent.be/webtools/plantcare/html/) was used to screen *cis*-acting elements in the promoter region. The gene function of the *GhNPF* family in *G. hirsutum* was annotated with gene ontology (GO) by using DAVID bioinformatics resources (https://david.ncifcrf.gov/). ChiPlot (https://www.chiplot.online/) online analytical tools was used to plot.

### Tissue-specific and abiotic stress-related expression profile analysis of *GhNPF* genes

To verify the expression profiles of the *GhNPF* genes in various tissues of *G. hirsutum*, the RNA-seq data for 9 tissue-specific samples of upland cotton (TM-1) (root, stem, leaf, sepal, petal, anther, pistil, ovule and fiber) and samples under salt, drought, cold and heat stress were downloaded from Zhejiang University (ZJU) (http://cotton.zju.edu.cn/) (Zhang et al., 2015). The *Gossypium* Resource and Network Database (GRAND) website (http://grand.cricaas.com.cn/home) was used to obtain the RNA-seq data for 9 different tissues of upland cotton (TM-1) and samples under salt, drought, cold, and heat stress from the CRI. The transcript abundance of *GhNPFs* in different tissues and in response to different abiotic stresses was calculated according to the fragments per kilobase of transcript per million mapped reads (FPKM) values. Heatmaps of all 98 *GhNPF* genes were generated using TBtools software, and Venn diagrams of candidate genes were plotted using the hiplot online website (https://hiplot-academic.com/basic/venn2).

### Experimental materials and stress exposure

The upland cotton cultivar Zhongmian 113 (ZM113) was grown in a greenhouse (25°C; 16 h/8 h light/darkness; humidity of approximately 60%-80%) at Gansu Agricultural University, Lanzhou, Gansu Province, China. The seeds were obtained from the CRI. Nine different organs (roots, stems, leaves, petals, sepals, anther, pistils, ovules and fibers) were collected from ZM113, which was healthy at budding and flowering stage and immediately frozen in liquid nitrogen for subsequent experiments. Healthy ZM113 plants of the same age (4 weeks old) were selected for abiotic stress treatments (heat, cold, salinity and drought). All the plants were grown in a growth chamber at 25°C before stress exposure. Each abiotic stress was applied for 0 h (control), 1 h, 3 h, 6 h, 12 h and 24 h (10 replications per treatment). Some ZM113 seedlings were subjected to cold (12°C) and heat (42°C) stress. For other ZM113 seedlings, their roots were soaked in 200 mmol/L NaCl and 15% polyethylene glycol (PEG-6000) to induce salinity and drought stresses. After the above stresses were applied, the shoot tips and young leaves were collected and immediately frozen in liquid nitrogen for subsequent experiments.

### qRT−PCR analysis of *GhNPFs*

Total RNA was extracted from the shoot tips and young leaf samples collected after the stress treatments and from the tissue of nine different organs *via* an RNA Prep Pure Plant Kit (Tiangen, China). Two micrograms of total RNA were used to synthesize 20 µl of cDNA using FastKing gDNA Dispelling RT SuperMix (KR118) (Tiangen, China) to analyze the relative expression of the *GhNPF* genes in the nine organs and under the different abiotic stresses. The *GhNPF* gene primers used were designed using NCBI Primer-BLAST (a primer design tool) and developed by Sangon Biotech (Shanghai) Co., Ltd.; the primers used are shown in **Table S1**. Real-time PCR amplification was performed using an LightCycler^®^ 96 Instrument together with SuperReal Premix Plus (SYBR Green) (FP209, Tiangen, China) according to the manufacturers’ instructions. The thermocycle procedure was as follows: 95°C for 3 minutes, followed by 40 cycles of 95°C for 5 seconds and 60°C for 15 seconds. All the data were normalized to those of actin (Wu et al., 2021), which served as an internal reference gene, and the relative expression of all the evaluated *GhNPF* genes was calculated using the 2^-ΔΔCt^ method (Willems et al., 2008). After normalization of the data from three independent experiments, all the data were expressed as the mean ± standard error. One-way analysis of variance (P<0.05), least significant difference (LSD) was used to evaluate the significance of each sample.

## Results

### Genome-wide identification and distribution of *NPF* family members in three *Gossypium* species

**Figure 1:**
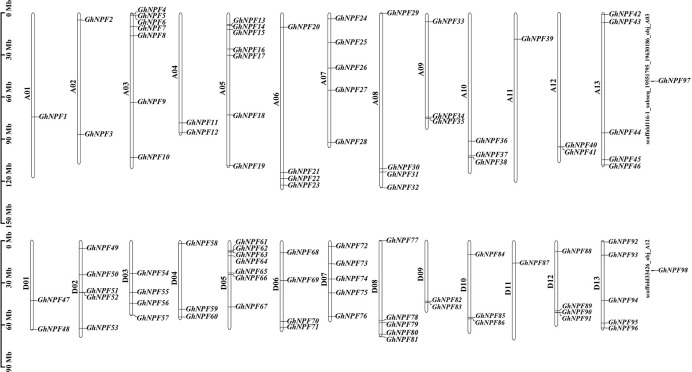
Positions of *GhNPFs* on the chromosomes of *G. hirsutum*. Partial *GhNPF* genes located on scaffolds. The white-colored bars show chromosomes from *the G. hirsutum* A_t_ and D_t_ subgenomes. A01-A13 and D01-D13 indicate chromosomes of the A_t_ and D_t_ subgenomes, respectively. The chromosomal positions of genes calculated from published genomic data are shown on the left side of each chromosome in the A_t_ and D_t_ subgenomes. The corresponding gene names are written on the right side of each chromosome of the A_t_ and D_t_ subgenomes.

In this study, in total, 98, 52 and 51 *NPF* genes were verified in *G. hirsutum*, *G. raimondii*, and *G. arboreum*, and the *G. hirsutum* genes were denoted *GhNPF1* to *GhNPF98* according to their physical locations on the chromosome (**Figure 1**). The details of these *GhNPF* gene family members and their related proteins are listed in **Table S2**. The interrelated protein length (amino acids [aa]) varied greatly from 537 aa (GhNPF18) to 818 aa (GhNPF81). The predicted molecular weights (MWs) and isoelectric points (pIs) of the proteins ranged from 59,756.73 Da (GhNPF19) to 89,983 Da (GhNPF79) and from 5.38 (GhNPF52) to 9.56 (GhNPF36), respectively. With respect to the secondary structure of the GhNPF proteins, alpha-helices (Hh) and random coils (Cc) accounted for a large proportion, while extended strands (Ee) and beta turns (Tt) constituted a comparatively low proportion. Subcellular localization predictions showed that the great majority of the proteins encoded by the *GhNPF* genes were located at the plasma membrane, except in the cases of those encoded by *GhNPF13* and *GhNPF61*.

The 96 *GhNPF* members were disproportionately located across the 26 chromosomes of *G. hirsutum*, and two genes (*GhNPF97* and *GhNPF98*) were on scaffolds (**Figure 1**). Chromosomes A03, A05 and D05 contained the greatest numbers of *GhNPFs* (7 members), while chromosomes A07, A13, D02, D07, D08 and D13 contained 5 *GhNPFs*, and they accounted for a large portion of the *GhNPFs* across the 26 chromosomes. In contrast, chromosomes A01, A11 and D11 contained the fewest *GhNPF* genes (1 member each).

### Gene duplication and collinearity analysis of *NPF* genes in *G. hirsutum*

**Figure 2:**
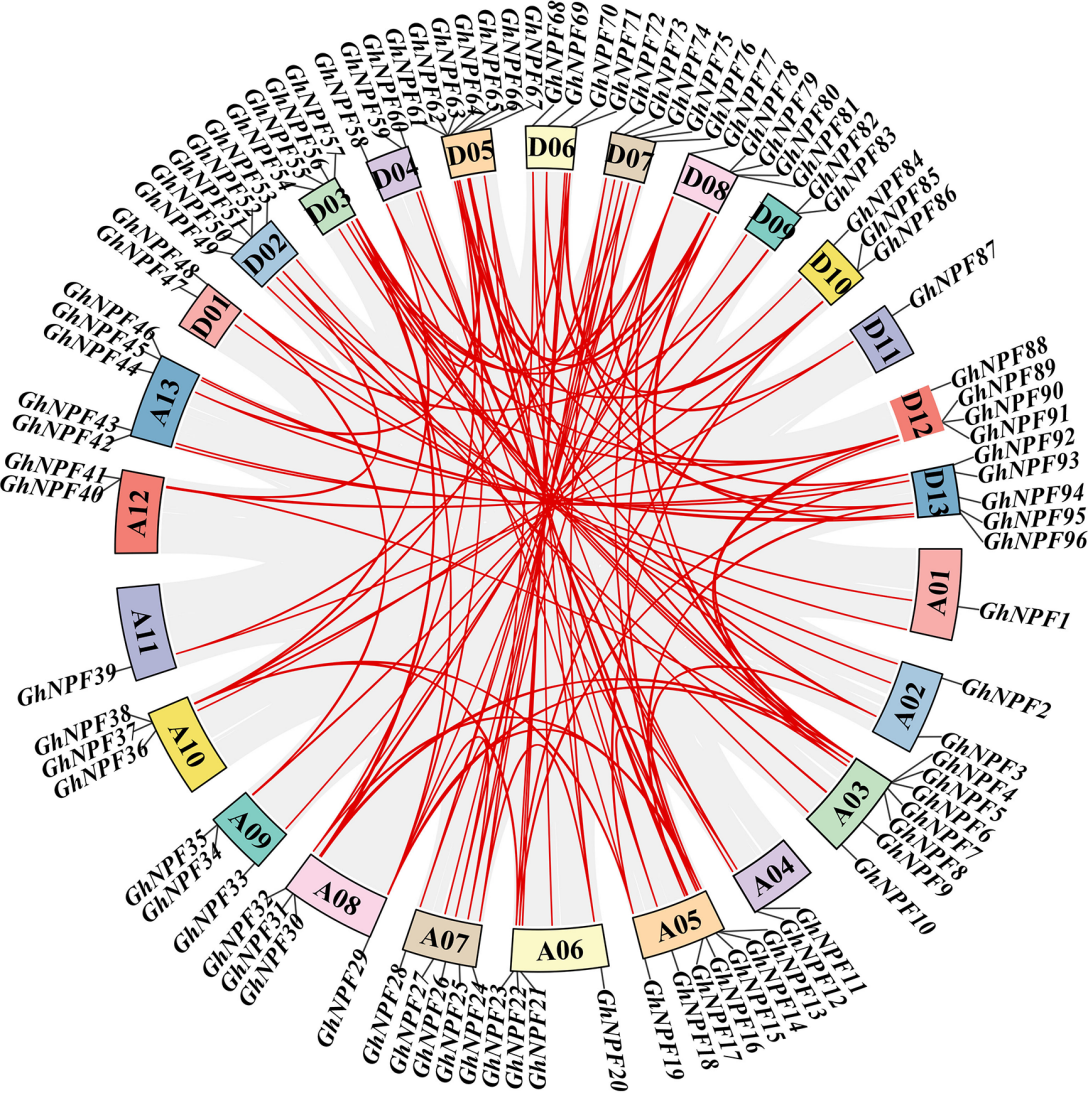
Duplication of *GhNPF* genes on chromosome 26 of *G. hirsutum*. The gray lines represent collinear relationships of all genes in the *G. hirsutum* genome, and the red lines represent gene pairs of *GhNPF*. The different colored rectangles indicate chromosomes.

To reveal the homologous locus relationships of the *GhNPF* gene family members between the A_t_ and D_t_ subgenomes in *G. hirsutum*, gene duplication events were studied using the MCScan tool to elucidate their amplification patterns. Two pairs of genes with tandem repeats were identified on chromosomes A03 and D05 (*GhNPF5/6* and *GhNPF63/64*), respectively. In addition, 84 segmentally duplicated genes were discovered in the *GhNPF* gene family of *G. hirsutum* (**Figure 2**, **Table S3**). These results showed that segmental duplication accounted for a large proportion of the evolution of the *GhNPF* gene family, which reflected the dominant role of segmental repeats relative to tandem repeats in the *GhNPF* gene family evolution. Moreover, the intergenomic synteny analysis results between *G. hirsutum* and two other *Gossypium* species were compared to further the understand homologous gene functions and phylogenetic relationships of *NPF* genes (**Figure S1**). The analysis of collinearity among the different species showed that 79 pairs of genes were collinear between *G. hirsutum* and *G. arboreum* and between *G. hirsutum* and *G. raimondii*. In conclusion, the present results provide evidence that *NPF* genes might undergo some genomic rearrangements during polyploidy. To better comprehend the evolutionary constraints controlling the functional divergence of the *GhNPF* gene family, the non-synonymous substitutions (Ka), synonymous substitutions (Ks), and non-synonymous to synonymous substitution (Ka/Ks) ratio were calculated (**Table S4**). All duplicated *GhNPF* gene pairs presented a Ka/Ks ratio of <1, suggesting that the *GhNPF* family genes might have experienced selective pressure throughout their evolution.

### Phylogenetic analysis of the *GhNPF* gene family

**Figure 3:**
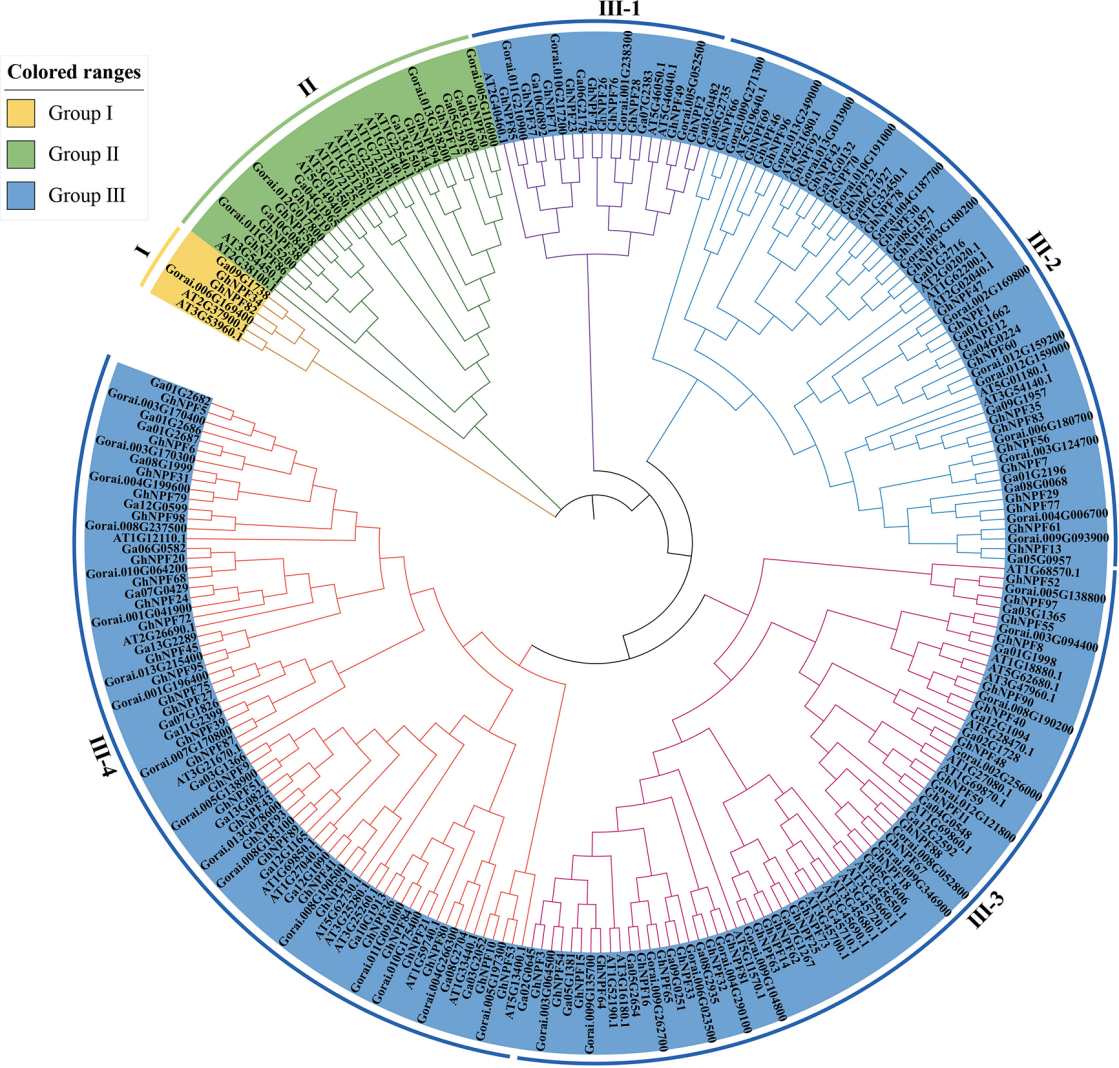
Phylogenetic tree of *NPF* genes in cotton and *Arabidopsis thaliana*. The tree was generated using the neighbor connection method of MEGA X software (1,000 bootstrap replicates). The tree was divided into three subfamilies, and the different colors show the following *NPF* subfamilies: yellow represents group I, green represents group II, and blue represents group III;. Group III was divided into four subgroups, in which different colors of branches represent different subgroups.

To analyze the phylogenetic relationships of the *NPFs* among *G. hirsutum, G. raimondii, G. arboreum* and *Arabidopsis*, a phylogenetic tree comprising the NPF proteins of *G. hirsutum* (n=98), *G. raimondii* (n=52), *G. arboreum* (n=51) and *Arabidopsis* (n=53) was constructed (**Figure 3**). The 98 GhNPF proteins clustered into three primary groups (Group I, Group II and Group III) according to bootstrap values (=1,000). There were only two *GhNPF* genes (*GhNPF34* and *GhNPF82*) in *G. hirsutum* belonging to Group I. There were eight *GhNPF* genes in *G. hirsutum* belonging to Group II. At the same time, Group III was unevenly divided into four subgroups: III-1, III-2, III-3 and III-4. Furthermore, the *GhNPF* members essentially clustered into subgroups III-2, III-3 and III-4, and the number of *GhNPFs* in *G. hirsutum* was two to three times greater than that in *Arabidopsis* among these subgroups. Among these species, 18 pairs of paralogous genes were found—15 pairs of genes in *Arabidopsis*, two pairs in *G. hirsutum* and one pair in *G. raimondii*. Furthermore, 86 pairs of orthologs from *G. hirsutum*, *G. arboreum* and *G. raimondii* were identified, revealing the paralogous and orthologous connections among these plant species.

### Structure and conserved motif analysis of *GhNPF* genes

**Figure 4:**
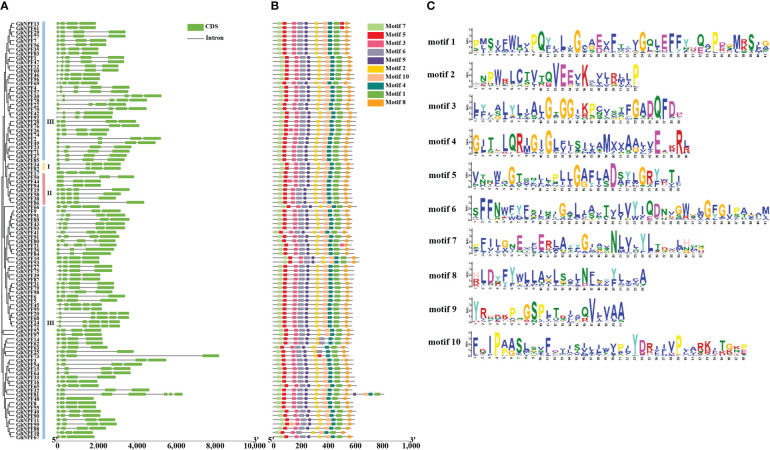
Phylogenetic relationships, structures and motif compositions of *GhNPF* genes. **(A)** Unrooted phylogenetic tree and exon/intron structure of *GhNPFs*. **(B)** Conserved motifs of 98 GhNPF proteins. **(C)** Conserved repeat markers of *GhNPF* genes.

To research the gene structure in the evolution of the *G. hirsutum* gene family, the structures of the *GhNPF* genes were obtained by analyzing the exon/intron boundaries (**Figure 4A**). The analysis of exon/intron structure revealed relatively high structural divergence among the *GhNPF* genes. The number of exons in the 98 *GhNPF* genes ranged from three to seven, and *GhNPF81* contained the most exons (n = 7). Most of the genes in Group I contained three introns, whereas in Group II, the genes contained two and four introns. Most of the genes in Group III contained three and four introns and the genes (*GhNPF81*) with the most introns were also included in the Group III. A total of 54.08% of all *GhNPFs* (53 genes) contained three introns each, suggesting that introns were gained and lost as the *GhNPF* gene family evolved, which might have resulted in functional diversity among the *GhNPF* genes.

Ten conserved motifs were detected in most GhNPF protein sequences by the use of the online MEME program, which further the similarities and differences in motif composition (**Figure 4B**). The amino acid numbers of the motifs ranged from 21 to 41 (**Figure 4C**). The number of motifs for each GhNPF was nine to fourteen (**Figures 4B, C**). Motifs 1, 2, 3, 4, 5, 6, 7 and 8 were present in all the GhNPF proteins, while motif 9 was not present only in the GhNPF18 protein of Group III. Similarly, motif 10 was not present in the GhNPF14, GhNPF62, GhNPF63, GhNPF25 and GhNPF73 proteins of Group III. In contrast, motifs 1-10 were all present in all the GhNPF members of Groups I and II. In general, almost all the GhNPF proteins within the same subgroup presented very similar motif compositions, suggesting that these GhNPF proteins have similar functions.

### Analysis of *cis*−acting elements of the *GhNPF* gene family

**Figure 5:**
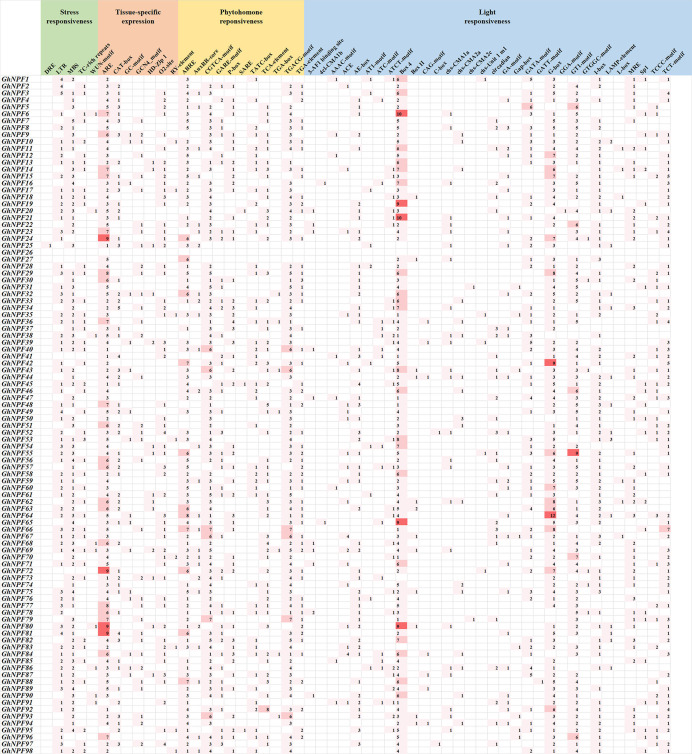
Prediction results of cis-regulatory elements in the promoter regions of *GhNPF* gene family members. The numbers in the cells represent the numbers of genes.

The *cis*-regulatory elements in the 2,000 bp upstream region of the 5’ end of the 98 *GhNPF* genes were identified and analyzed to reveal their potential response mechanisms (**Figure 5**). We identified 55 *cis*-regulatory elements involved in stress responsiveness, tissue-specific expression, phytohormone responsiveness and light responsiveness. Five stress-related elements were identified, namely, DREs, LTRs, MBSs, TC-rich repeats and WUN motif–containing elements. These *cis*-acting elements were involved in responses to low temperature, salt stress, defense and drought. There were seven *cis*-acting elements associated with tissue-specific expression, namely, AREs, CAT-boxes, GC-motif, GCN4_motif, HD-Zip 1s, O2-site–and RY elements. Moreover, among these elements AREs were the most common in the *GhNPF* gene promoters. In addition, eleven hormone-related elements, namely, ABREs, AuxRR-core–containing elements, CGTCA motif–containing elements, GARE motif–containing elements, P-boxes, SAREs, TATC-boxes, TCA elements, TGA-boxes, TGACG motif–containing elements and TGA elements, were also found. This category included abscisic acid-responsive elements (ABREs), auxin-responsive elements (AuxRR-core–containing elements, TGA-boxes and TGA motif–containing elements), methyl jasmonate (MeJA)-responsive elements (CGTCA motif–containing elements and TGACG motif–containing elements), gibberellin-responsive elements (GARE motif–containing elements, P-boxes and TATC motif–containing elements) and salicylic acid-response elements (SAREs and TCA elements). There were also 32 *cis*-acting elements related to the light response, including Box 4 elements, C-boxes, G-boxes, etc. Box 4 elements and G-boxes were present in relatively high numbers within the light-responsive *cis*-acting regulatory elements. Interestingly, we found that the *GhNPF26* gene does not contain any type of *cis*-acting element. Taken together, these results showed that *GhNPF* genes might play an important role in abiotic stress responses, defense-related signal transduction, and phytohormone responses. In addition, the genes might be involved in various light responses during *G. hirsutum* growth.

### Expression patterns of *GhNPF* genes in different tissues and gene ontology

**Figure 6:**
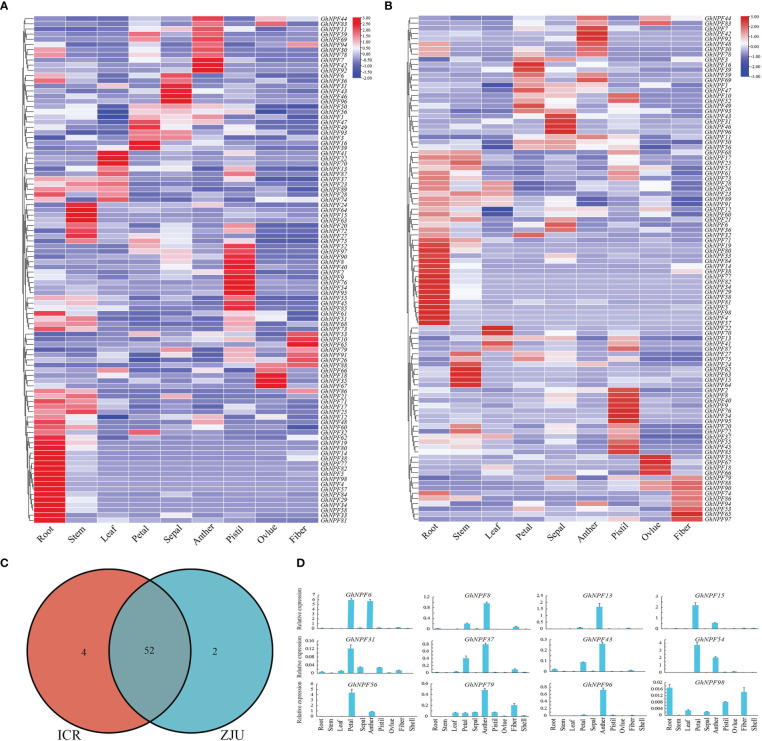
Expression profiles of *GhNPF* genes in 9 organs of upland cotton. **(A, B)** The expression patterns of 98 *GhNPFs* in 9 *G*. *hirsutum* (TM-1) tissues were analyzed by RNA-seq at the ZJU and CRI. The red and purple colors indicate high and low expression levels, respectively. **(C)** Venn diagram of common highly expressed genes in the two RNA-seq datasets (ZJU and CRI). **(D)** Relative expression levels of 14 *GhNPFs* in 9 organs of *G*. *hirsutum*. The error bars represent the standard deviations of three biological replications.

To analyze the expression patterns of *GhNPF* genes during *G. hirsutum* development, RNA-seq data of various *G. hirsutum* tissues were used in this study. The expression characteristics of all 98 *GhNPF* genes were determined at varying levels across different tissues and developmental stages (**Figure 6**). The RNA-seq data of ZJU showed that 55.1% of *GhNPF* genes were highly expressed in vegetative organs (roots, stems and leaves) and 65.3% of *GhNPF* genes were highly expressed in reproductive organs (petals, sepals, anther, pistils, ovules and fibers). In addition, according to the FPKM value of CRI’s RNA-seq data, 56 out of 98 *GhNPF* genes were highly expressed in vegetative organs (roots, stems and leaves), and 71 out of 98 *GhNPF* genes were highly expressed in reproductive organs (petals, sepals, anther, pistils, ovules and fibers). A total of 52 common genes were identified in vegetative organs from two RNA-seq datasets, which verified the reliability of the data (**Figure 6C**). The expression of 14 select genes in the tissues of *G. hirsutum* was examined *via* qRT−PCR, and the results were essentially consistent with the RNA-seq data (**Figure 6D**).

**Figure 7:**
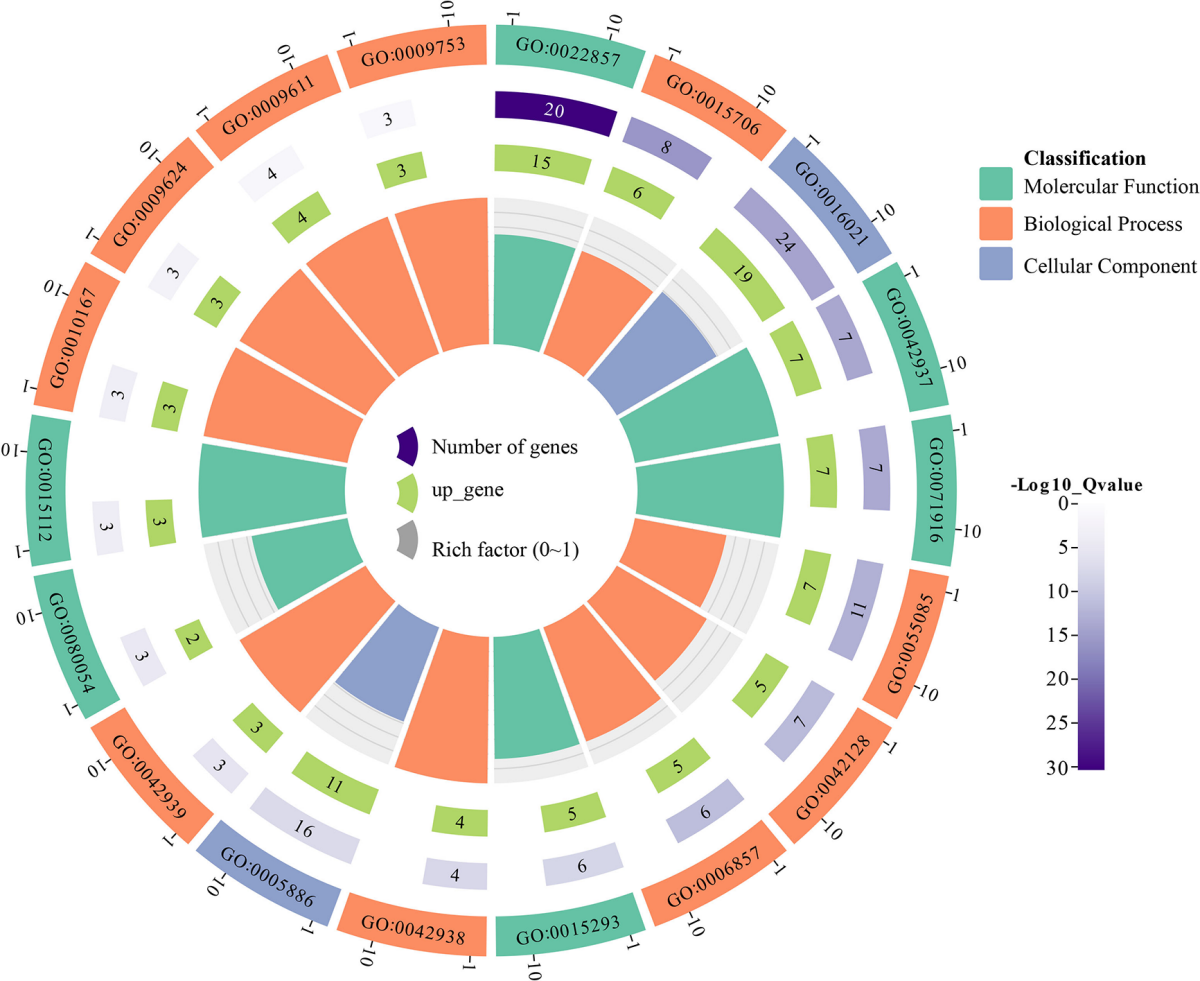
Functional categorization of the *GhNPF* genes in *G. hirsutum*. Purple represents the number of genes, light green represents the number of upregulated genes, gray represents the rich factor, green represents molecular function, orange represents biological process, and light purple represents cellular component.

To further understand the functional segregation of the identified *GhNPF* genes, GO was performed by DAVID based on three categories: molecular function, biological process and cellular component (**Figure 7**). A total of 39 *GhNPF* genes belonged to molecular function, including transmembrane transporter activity (GO:0022857), tripeptide transporter activity (GO:0042937), dipeptide transmembrane transporter activity (GO:0071916), symporter activity (GO:0015293), low-affinity nitrate transmembrane transporter activity (GO:0050054) and nitrate transmembrane transporter activity (GO:0015112). Twenty-six *GhNPF* genes were involved in nitrate transport (GO:0015706), transmembrane transport (GO:0055085), nitrate assimilation (GO:0042128), oligopeptide transport (GO:0006857), dipeptide transport (GO:0042938), tripeptide transport (GO:0042939), response to nitrate (GO:0010167), response to nematode (GO:0009624), response to wounding (GO:0009611) and response to jasmonic acid (GO:0009753) in biological process. A total of 24 genes can function as an integral component of the membrane (GO:0016021) and plasma membrane (GO:0005886) in cellular component. Interestingly, some *GhNPFs* exist in different cell components, participate in different biological processes, and have multiple molecular functions.

### Expression of *GhNPF* genes in response to abiotic stresses

**Figure 8:**
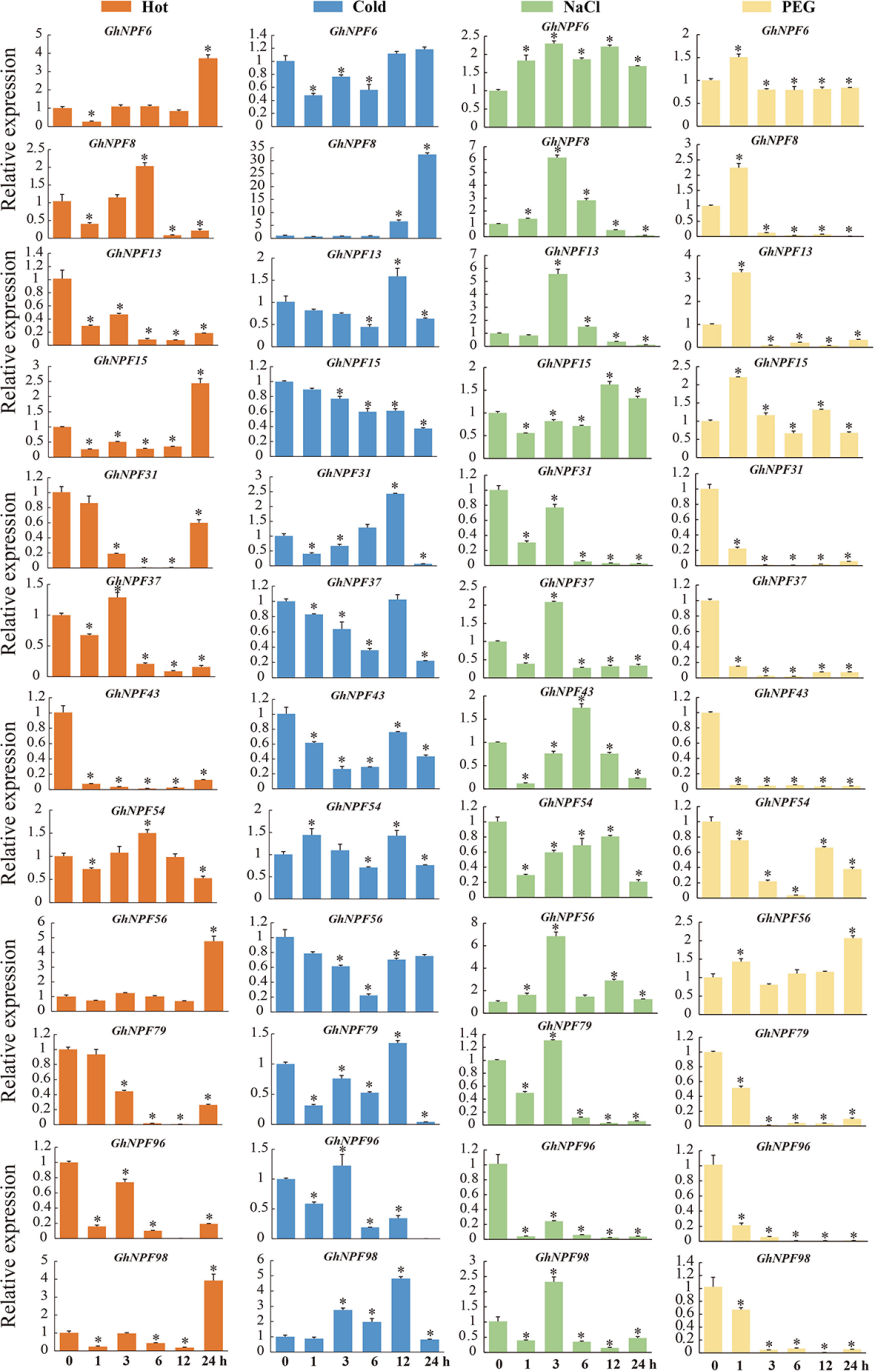
Relative expression levels of 12 *GhNPFs* in response to cold, salt, and drought treatments. The error bars represent the standard deviations of three biological replications. Orange represents heat stress, blue represents cold stress, green represents salt stress and yellow represents drought stress. Asterisks were used to indicate a significant degree of expression compared to the value of the control (*P < 0.05).

To analyze the potential functions of the *GhNPF* genes in response to abiotic stresses, the *GhNPF* expression levels were evaluated *via* two RNA-seq datasets (those compiled by ZJU and the ICR) corresponding to plants under salt, drought, cold and heat treatments. Analysis of the expression profiles showed that six genes, namely, *GhNPF5*, *GhNPF16*, *GhNPF18*, *GhNPF65*, *GhNPF69* and *GhNPF86*, were not expressed in any of the four treatments; moreover, *GhNPF77* and *GhNPF29* were not expressed in response to temperature stress, and *GhNPF88* was not expressed in response to drought and salt stress (**Figure S2**). The expression of several genes was significantly increased or decreased under the cold, heat, NaCl and PEG treatments compared with the control treatment, and the DEGs differed at different treatment time periods. According to the FPKM values of the ZJU RNA-seq dataset, 52, 36, 41 and 48 genes were highly expressed during at least two of the five different time periods (1 h, 3 h, 6 h, 12 h and 24 h) and stress treatment groups (heat, cold, salt and drought) (**Figures S3A, C**). According to the FPKM values of the CRI RNA-seq dataset, 51, 36, 51 and 31 genes were highly expressed in at least two of the five different time stress treatment groups (heat, cold, salt and drought, respectively) (**Figures S3A, C**). These results showed that after heat, cold, salt and drought treatment, 59 (heat and cold) and 38 (salt and drought) genes were upregulated, and according to the two datasets (**Figures S3B, E**), 31 genes common to the temperature (heat and cold) and saline-alkali (salt and drought) treatments could be candidate genes with resistance-related characteristics in cotton (**Figure S3F**). Among these candidate genes, the *GhNPF6*, *GhNPF37* and *GhNPF54* genes were all upregulated after heat and cold treatment, but the expression of the *GhNPF64* and *GhNPF27* genes was inhibited by the temperature treatments. In addition, the *GhNPF28*, *GhNPF55* and *GhNPF78* genes were all upregulated after NaCl and PEG treatment, while the *GhNPF74* gene was upregulated only after NaCl treatment; PEG treatment inhibited the expression of the *GhNPF39* gene. To further investigate the possible response of *GhNPF*s to abiotic stress conditions, by performing qRT−PCR, we analyzed the expression of 13 select genes from different tissues of *G. hirsutum* under different stresses (**Figure 8**). The heat treatments (at all time points) induced the expression of *GhNPF13*, *GhNPF31*, *GhNPF43*, *GhNPF79* and *GhNPF96*, and cold stress exposure at all time intervals induced the expression of *GhNPF15*, *GhNPF43* and *GhNPF56*. Similarly, the salinity stress treatments induced the expression of *GhNPF6*, *GhNPF31*, *GhNPF54*, *GhNPF57* and *GhNPF96* at all time intervals, and the *GhNPF31*, *GhNPG37*, *GhNPF43*, *GhNPF79*, *GhNPF96* and *GhNPF98* genes exhibited increased expression in response to drought stress. Taken together, the results of our abiotic stress response gene expression analysis showed that the *GhNPF* gene family members in upland cotton have potential regulatory roles in the response to abiotic stress.

## Discussion

N plays a substantial role in the growth and development of plants under abiotic stresses (Ercoli et al., 2010; Zhang et al., 2014; Goel and Singh, 2015; Taochy et al., 2015). *NPFs* are LATSs of N or NO_3_^-^ and compose the largest subfamily of NO_3_^-^ transporters in plants (O’Brien et al., 2016; Fan et al., 2017). The *NPF* family members plant species and subspecies such as *Arabidopsis*, *Populus*, rice, *Brassica napus*, soybean, *Brassica rapa* subsp. *pekinensis*, *Populus tomentosa* and *P. trifoliata* were identified and analyzed to determine their gene structure and transcript accumulation (Tsay et al., 2007; Bai et al., 2013; Drechsler et al., 2018; Zhang et al., 2020; You et al., 2020; Ma et al., 2021; Zhao et al., 2021; Zhao et al., 2022). In this study, 99, 52 and 51 *NPF* genes were identified in *G. hirsutum*, *G. raimondii*, and *G. arboreum*, respectively, compared to other identified plant species. Fifty-three have been identified in *Arabidopsis* (Tsay et al., 2007), along with 68 in *Populus* (Bai et al., 2013), 82 in rice (Drechsler et al., 2018), 193 in *B. napus* (Zhang et al., 2020), 120 in soybean (You et al., 2020), 72 in *B. rapa* subsp. *pekinensis* (Ma et al., 2021), 87 in *P. tomentosa* (Zhao et al., 2021) and 56 in *P. trifoliata* (Zhao et al., 2022). The number of genes in *G. hirsutum* was similar to the number of genes in *P. tomentosa* and was twice that in *Arabidopsis*. Genome-wide identification of the *NPF* genes in cotton was conducted to analyze the phylogenetic relationships of the *NPFs* between *G. hirsutum* and two other cotton species as well as *A. thaliana*. The NPF proteins could be separated into three main groups, namely, I, II, and III, according to the phylogenetic results, of which Group III could be further divided into four subgroups: III-1, III-2, III-3, and III-4. In these species, 18 pairs of paralogous genes were found: there were 15 pairs of genes in *Arabidopsis*, two pairs in *G. hirsutum* and one pair in *G. raimondii*. Furthermore, 86 pairs of orthologs from *G. hirsutum*, *G. arboreum* and *G. raimondii* were identified, suggesting that polyploidy led to the evolution of new cotton-specific ortholog clusters. During long-term natural selection, basic *NPF* genes were retained in the *G. hirsutum* genome, while others were lost, which is consistent with the findings of a study involving *B. napus* (Zhang et al., 2020). Other studies have shown that genes within the same taxa might have similar functions due to sequence similarity (Nan et al., 2021). Analysis of the exon/intron structure revealed a relatively high structural divergence among the *GhNPF* genes. The results suggested that events in which introns were lost and gained occurred during the evolution of the *GhNPF* gene family, which might result in functional redundancy among *GhNPF* genes. Therefore, cotton *NPF* family members might have differentiated during evolution, which might have resulted in functional differences.

Gene duplication is the main mechanism through which gene families expand. Segmental and tandem duplication are considered to be the two main causes of gene family expansion in plants (Cannon et al., 2004). The number of segmental duplication of *GhNPFs* in *G. hirsutum* was lower than that in *B. napus* (Zhang et al., 2020), and 84 segmentally duplicated genes were discovered in the *GhNPF* gene family of *G. hirsutum*, while the number of tandemly duplicated genes of both species was the same. Nevertheless, collinearity analysis of different species is one way to study the gene evolution and relationships (Yu et al., 2020). Therefore, the results of the intergenomic synteny analyses between *G. hirsutum* and the other two cotton species were compared to further understand the homologous gene functions and phylogenetic relationships of the *NPF* genes. The results showed that since the number of *G. hirsutum* genes was slightly greater than the total number of *G. arboreum* and *G. raimondii* genes, compared with those in *G. hirsutum*, the *NPF* gene duplication events and chromosomal rearrangements in *G. arboreum* and *G. raimondii* might be conserved. Likewise, duplication events in the *B. napus* genome might have facilitated the expansion of the *NPF* gene family (Zhang et al., 2020). Generally, due to the high diversity and allopolyploid characteristics of the *NPF* gene family, the members of the *NPF* gene family might have complex phylogenetic relationships in *G. hirsutum*. In order to investigate differentiation after gene duplication, non-synonymous substitutions (Ka) and synonymous substitutions (Ks) of replicated *GhNPF* genes in *G. hirsutum* were calculated. The present results suggested that *GhNPF* family genes have experienced selective pressures during evolution.

*Cis*-acting regulatory elements play paramount roles in regulating gene transcription by coordinating responses to developmental and environmental cues (Schmitz et al., 2022). It has been found that *NPF* transport is affected by nitrite, auxin, abscisic acid, jasmonoyl-isoleucine, and gibberellins, and *NPF* transport even participates in flowering time regulation and is negatively affected by abiotic stresses (Sugiura et al., 2007; Krouk et al., 2010; Kanno et al., 2012; Chiba et al., 2015; Goel and Singh, 2015; Saito et al., 2015; David et al., 2016; Teng et al., 2019). In this study, 55 types of *cis*-acting elements (stress-responsive, tissue-specific, phytohormone-responsive and light-responsive ones) were confirmed in the promoters of *GhNPF*s. Most *GhNPF* genes contained stress-responsive elements, hormone-responsive elements and light-responsive elements, which indicated that the expression and regulation of these genes were affected by stress, hormones and light. Like in the *P. trifoliata* study (Zhao et al., 2022), in the present study, the *GhNPF* promoters contained MyB-binding sites, indicating that these genes might be regulated by the same transcriptional mechanism. There is direct evidence that *NPF* genes are affected by salt and drought stress (Zhang et al., 2014). Furthermore, based on two RNA-seq datasets and qRT−PCR analyses, we characterized the spatial and temporal expression profiles of *NPF* genes and the responses of *NPF* genes to various stress treatments in *G. hirsutum* and found that a large number of *GhNPF* genes were highly expressed in the roots, stems and pistils, suggesting that *GhNPF* might be important for the functions of those organs. *GhNPF37* was expressed in all the tested tissues, while *GhNPF5*, a member of the NPF6 family, was mainly expressed in the roots, and *AtNPF6.3* was also highly expressed in the lateral roots (Guo et al., 2001). Both *GhNPF56* and *GhNPF13* are members of the NPF8 family and were highly expressed in the petals. However, *AtNPF8.2* is mainly expressed in the pollen and ovules (Komarova et al., 2008). *GhNPF* family members were unevenly expressed across all the evaluated tissues, indicating that they played an important role in controlling the growth and development of *G. hirsutum*. Interestingly, some *GhNPFs* exist in different cell components, participate in different biological processes, and have multiple molecular functions. This gene family may play an important role in the growth process and environmental diversity. For example, *GhNPF6* is located in the plasma membrane, has membrane boundary functions such as transmembrane transporter activity, and participates in nitrogen compound transport, response to nitrate, response to wounding and response to jasmonic acid. Research has shown that *NPF* genes respond specifically to abiotic and biotic stressors except N starvation (Fan et al., 2017). For example, *GsNRT1.12*, *GsNRT1.43*, *GsNRT1.62*, and *GsNRT1.57* in soybean were shown to be rapidly upregulated after salt treatment (You et al., 2020). *Phyllostachys edulis* responds to cold and drought treatment through altered expression of *PeNPF* to a certain extent (Yuan et al., 2021). To further investigate the potential functions of *GhNPFs* in abiotic stress responses, we analyzed the gene expression profile data. In addition, qRT−PCR was used to analyze the expression of 13 *GhNPFs* under four abiotic stress conditions: salt, drought, heat and cold. Two genes (*GhNPF31* and *GhNPF96*) were downregulated after three treatments, namely, heat, salinity and drought, and the expression of *GhNPF31* was the highest after 12 h of cold treatment, while the expression of *GhNPF96* was the highest after 3 h of cold treatment, the results of which implied that *GhNPFs* might participate in the transduction of different signaling pathways in response to abiotic stress. The expression level of *PtrNPF7.3* in *P. trifoliata* (Zhao et al., 2022), a homologous gene of *GhNPF96*, was lower in the control group than in the treatment groups, but its transcript level significantly increased after salt treatment. The *GhNPF6* gene was upregulated under salt, but cold, heat and drought had little effect on its expression. The expression of the *GhNPF79* and *GhNPF98* genes was downregulated in response to drought treatment, and their transcript levels increased after 3 h of salt treatment then began to decrease at 6 h, 12 h and 24 h. Similarly, the expression level of *NRT1.1*, a homolog of *GhNPF79* and *GhNPF98*, was reduced in *B. juncea* and *Arabidopsis* after salt and drought stresses (Goel and Singh, 2015; Taochy et al., 2015). High temperature inhibited the expression of 79 genes under heat at 24 h. Interestingly, *GhNPF5* was significantly expressed in the roots but not in response to the four biotic stresses, which proved that the *GhNPF* gene responds to specific stressors, which explains why it was not expressed under any one stress. These results suggested that NO_3_^-^ uptake might increase the osmotic potential of cells in response to abiotic stress. In general, this study revealed the *NPF* genes in *G. hirsutum* and explored their expression profiles in different tissues and under different abiotic stresses, the findings of which provide a theoretical basis for further studies on the function of *GhNPFs* and plant N use efficiency under abiotic stress.

## Data availability statement

The datasets presented in this study can be found in online repositories. The names of the repository/repositories and accession number(s) can be found in the article/Supplementary Material.

## Author contributions

JL, CW and JS designed the research. JL, JJ and YL performed the experiments. CL and JP analyzed the data. JL wrote the manuscript. CW and JS revised the manuscript. All authors contributed to the article and approved the submitted version.
