# Supplementary material for: Genome-wide investigation and expression profiles of the NPF gene family provide insight into the abiotic stress resistance of Gossypium hirsutum
Source: Front Plant Sci. 2023 Jan 19;14:1103340. doi: 10.3389/fpls.2023.1103340 (PMC9893419; doi:10.3389/fpls.2023.1103340)
Supplement: Supplementary file 1 [file DataSheet_1.docx]

Supplementary Material

# Supplementary Tables and Figures

## Supplementary Tables

Table S1 Sequences of the primer pairs used in the qRT-PCR analysis of candidate genes.

| **Gene** | **Sense primer (5'–3')** | **Antisense primer (5'–3')** |
| --- | --- | --- |
| *GhNPF5* | CCGTTCAAGCAACTGGTGTC | AGTCCCAAGGGCTATGAGGT |
| *GhNPF6* | ATTCGATGATTCGGACCCCG | CATAGCCCCATTCACGTCCT |
| *GhNPF8* | GGGTTTAGCCATTCCTGCCT | TGTGATTGGGCTACCTTGGG |
| *GhNPF13* | GTTTCTTCGACAAAGCGGCA | TTTCAGCTCCTCGACCTGTG |
| *GhNPF15* | CTATGGGCTGGTTCCCAACA | ATTGGTAGCAGCTTGCCAGA |
| *GhNPF31* | GCTCTGTCTTCTCGGTGGTT | GAGTTGTATGCCGCTTGCTG |
| *GhNPF37* | CATGCGTCGAAAAACCGGAA | TCGAACCTCGACTGCGTATG |
| *GhNPF43* | AGAGCTGTGGTGTACAAGCC | TGAGCTAGGCAGCAGTTGAG |
| *GhNPF54* | ATTCCCGCAGGCTCTTATGG | CTGCCATAGCCAAGCAGGTA |
| *GhNPF56* | CATGGCATCAAGCCAAGGTG | TTCTCGGTCTCGTTGGTGTC |
| *GhNPF79* | TGAGAACCGCAAGCTCACAT | TTCATCCCTTTGGGGCACTC |
| *GhNPF96* | GCAGCGTTCTCATTTGGACC | GACACGGATTTGAGCCGTTG |
| *GhNPF98* | GATTCTAGGTGGCGAGGCAT | GCCACCGAGTAGACACAACA |

Table S2 Detailed information on the *GhNPF* gene family members in *Gossypium hirsutum*.

| **Gene ID** | **Gene name** | **aa** | **Mw (Da)** | **pI** | **Hh** | **Ee** | **Tt** | **Cc** | **Subcellular location** |
| --- | --- | --- | --- | --- | --- | --- | --- | --- | --- |
| GH_A01G1530 | *GhNPF1* | 584 | 64678.78 | 5.97 | 46.75% | 12.67% | 3.94% | 36.64% | plasma membrane |
| GH_A02G0439 | *GhNPF2* | 594 | 66292.32 | 9.16 | 51.85% | 13.13% | 3.70% | 31.31% | plasma membrane |
| GH_A02G1421 | *GhNPF3* | 591 | 65153.56 | 9.36 | 50.08% | 13.37% | 4.06% | 32.49% | plasma membrane |
| GH_A03G0072 | *GhNPF4* | 600 | 65415.31 | 9.04 | 45.00% | 14.00% | 3.67% | 37.33% | plasma membrane |
| GH_A03G0138 | *GhNPF5* | 585 | 64540.6 | 9.05 | 47.35% | 12.99% | 2.91% | 36.75% | plasma membrane |
| GH_A03G0140 | *GhNPF6* | 585 | 66617.21 | 7.03 | 48.55% | 13.33% | 3.42% | 34.70% | plasma membrane |
| GH_A03G0609 | *GhNPF7* | 570 | 62638.26 | 9.23 | 45.96% | 13.68% | 3.68% | 36.67% | plasma membrane |
| GH_A03G0780 | *GhNPF8* | 590 | 64130.31 | 8.74 | 46.61% | 12.37% | 3.22% | 37.80% | plasma membrane |
| GH_A03G1176 | *GhNPF9* | 570 | 64781.98 | 8.96 | 49.82% | 12.46% | 2.63% | 35.09% | plasma membrane |
| GH_A03G1757 | *GhNPF10* | 634 | 70503.17 | 8.91 | 47.16% | 11.99% | 2.68% | 38.17% | plasma membrane |
| GH_A04G1174 | *GhNPF11* | 602 | 64521.69 | 5.73 | 45.85% | 14.12% | 3.49% | 36.54% | plasma membrane |
| GH_A04G1524 | *GhNPF12* | 584 | 66729.97 | 9.21 | 46.75% | 12.50% | 4.62% | 36.13% | plasma membrane |
| GH_A05G0912 | *GhNPF13* | 570 | 62338.24 | 8.16 | 45.96% | 14.04% | 3.33% | 36.67% | nuclear |
| GH_A05G1017 | *GhNPF14* | 577 | 65951.08 | 8.45 | 45.58% | 12.65% | 3.81% | 37.95% | plasma membrane |
| GH_A05G1317 | *GhNPF15* | 580 | 63216.06 | 6.92 | 46.90% | 13.97% | 3.62% | 35.52% | plasma membrane |
| GH_A05G2485 | *GhNPF16* | 601 | 63897.91 | 9.13 | 46.92% | 13.98% | 3.99% | 35.11% | plasma membrane |
| GH_A05G2751 | *GhNPF17* | 571 | 64235.37 | 8.73 | 47.46% | 12.61% | 3.50% | 36.43% | plasma membrane |
| GH_A05G3381 | *GhNPF18* | 537 | 63745.48 | 9.12 | 49.53% | 13.59% | 3.54% | 33.33% | plasma membrane |
| GH_A05G4208 | *GhNPF19* | 578 | 59756.73 | 8.44 | 48.79% | 12.98% | 3.29% | 34.95% | plasma membrane |
| GH_A06G0613 | *GhNPF20* | 586 | 64015.57 | 9.22 | 48.81% | 13.82% | 2.39% | 34.98% | plasma membrane |
| GH_A06G1686 | *GhNPF21* | 577 | 63857.76 | 9.12 | 50.95% | 9.88% | 2.95% | 36.22% | plasma membrane |
| GH_A06G1827 | *GhNPF22* | 597 | 65183.66 | 9.02 | 48.91% | 11.73% | 3.35% | 36.01% | plasma membrane |
| GH_A06G2054 | *GhNPF23* | 583 | 65857.33 | 8.16 | 47.68% | 14.41% | 3.77% | 34.13% | plasma membrane |
| GH_A07G0410 | *GhNPF24* | 581 | 68038.29 | 9.16 | 48.88% | 12.39% | 3.44% | 35.28% | plasma membrane |
| GH_A07G1265 | *GhNPF25* | 558 | 61213.99 | 8.61 | 48.03% | 13.44% | 3.41% | 35.13% | plasma membrane |
| GH_A07G1648 | *GhNPF26* | 610 | 67586.87 | 8.67 | 49.67% | 12.13% | 3.28% | 34.92% | plasma membrane |
| GH_A07G1816 | *GhNPF27* | 595 | 63453.47 | 9.06 | 49.92% | 12.77% | 3.03% | 34.29% | plasma membrane |
| GH_A07G2319 | *GhNPF28* | 609 | 65747.83 | 8.95 | 51.07% | 13.79% | 2.79% | 32.35% | plasma membrane |
| GH_A08G0060 | *GhNPF29* | 576 | 64160.62 | 8.05 | 47.74% | 13.02% | 4.34% | 34.90% | plasma membrane |
| GH_A08G1847 | *GhNPF30* | 599 | 64511.68 | 9.21 | 46.91% | 12.85% | 3.84% | 36.39% | plasma membrane |
| GH_A08G1961 | *GhNPF31* | 583 | 64192.07 | 7.89 | 47.00% | 14.92% | 3.09% | 34.99% | plasma membrane |
| GH_A08G2837 | *GhNPF32* | 583 | 66570.06 | 6.24 | 47.00% | 14.92% | 3.09% | 34.99% | plasma membrane |
| GH_A09G0240 | *GhNPF33* | 603 | 66070.49 | 8.65 | 45.61% | 15.26% | 4.48% | 34.66% | plasma membrane |
| GH_A09G1695 | *GhNPF34* | 594 | 66053.23 | 8.6 | 47.81% | 13.97% | 3.03% | 35.19% | plasma membrane |
| GH_A09G1796 | *GhNPF35* | 569 | 63838.12 | 8.6 | 46.05% | 14.59% | 3.69% | 35.68% | plasma membrane |
| GH_A10G1730 | *GhNPF36* | 597 | 64022.75 | 9.56 | 51.09% | 11.22% | 2.18% | 35.51% | plasma membrane |
| GH_A10G1981 | *GhNPF37* | 583 | 66187.95 | 8.6 | 48.71% | 13.55% | 2.92% | 34.82% | plasma membrane |
| GH_A10G2024 | *GhNPF38* | 584 | 65173.62 | 8.91 | 48.29% | 12.84% | 3.60% | 35.27% | plasma membrane |
| GH_A11G1605 | *GhNPF39* | 595 | 65920.28 | 9.11 | 51.93% | 13.95% | 3.19% | 30.92% | plasma membrane |
| GH_A12G1936 | *GhNPF40* | 611 | 68068.75 | 9.28 | 49.43% | 10.97% | 2.78% | 36.82% | plasma membrane |
| GH_A12G1939 | *GhNPF41* | 551 | 60734.32 | 8.79 | 53.72% | 10.89% | 2.72% | 32.67% | plasma membrane |
| GH_A13G0128 | *GhNPF42* | 616 | 61461.7 | 8.01 | 45.94% | 13.15% | 3.41% | 37.50% | plasma membrane |
| GH_A13G0524 | *GhNPF43* | 580 | 64061.06 | 8.94 | 49.48% | 12.07% | 2.93% | 35.52% | plasma membrane |
| GH_A13G1419 | *GhNPF44* | 562 | 63764.33 | 8.54 | 51.96% | 10.50% | 3.74% | 33.81% | plasma membrane |
| GH_A13G2117 | *GhNPF45* | 579 | 65389.68 | 6.05 | 48.36% | 14.85% | 2.07% | 34.72% | plasma membrane |
| GH_A13G2425 | *GhNPF46* | 598 | 68492.32 | 6.28 | 50.67% | 12.88% | 3.34% | 33.11% | plasma membrane |
| GH_D01G1628 | *GhNPF47* | 584 | 63078.29 | 9.22 | 45.21% | 13.87% | 3.42% | 37.50% | plasma membrane |
| GH_D01G2465 | *GhNPF48* | 569 | 64610.7 | 5.73 | 47.10% | 12.48% | 3.16% | 37.26% | plasma membrane |
| GH_D02G0451 | *GhNPF49* | 594 | 70646.26 | 8.71 | 50.17% | 12.79% | 3.37% | 33.67% | plasma membrane |
| GH_D02G1056 | *GhNPF50* | 573 | 66315.4 | 9.16 | 50.09% | 11.69% | 3.84% | 34.38% | plasma membrane |
| GH_D02G1242 | *GhNPF51* | 570 | 65818.38 | 8.37 | 50.53% | 11.75% | 2.81% | 34.91% | plasma membrane |
| GH_D02G1243 | *GhNPF52* | 593 | 62458.34 | 5.38 | 48.06% | 11.64% | 2.36% | 37.94% | plasma membrane |
| GH_D02G1913 | *GhNPF53* | 634 | 62629.26 | 9.21 | 46.21% | 13.56% | 3.00% | 37.22% | plasma membrane |
| GH_D03G0790 | *GhNPF54* | 591 | 64789.84 | 9.08 | 48.05% | 14.55% | 4.06% | 33.33% | plasma membrane |
| GH_D03G1042 | *GhNPF55* | 590 | 66547.96 | 6.29 | 44.24% | 12.71% | 3.90% | 39.15% | plasma membrane |
| GH_D03G1347 | *GhNPF56* | 570 | 65542.46 | 8.8 | 47.02% | 13.68% | 3.86% | 35.44% | plasma membrane |
| GH_D03G1892 | *GhNPF57* | 600 | 65181.5 | 9.43 | 47.33% | 13.17% | 4.00% | 35.50% | plasma membrane |
| GH_D04G0171 | *GhNPF58* | 578 | 64522.67 | 5.6 | 49.31% | 12.98% | 2.94% | 34.78% | plasma membrane |
| GH_D04G1513 | *GhNPF59* | 602 | 63935.6 | 9.07 | 45.02% | 12.29% | 2.49% | 40.20% | plasma membrane |
| GH_D04G1867 | *GhNPF60* | 584 | 66684.9 | 9.27 | 45.21% | 12.84% | 3.77% | 38.18% | plasma membrane |
| GH_D05G0904 | *GhNPF61* | 570 | 63206.01 | 6.67 | 46.67% | 13.33% | 3.86% | 36.14% | nuclear |
| GH_D05G1004 | *GhNPF62* | 577 | 63710.65 | 9.13 | 45.75% | 13.00% | 3.99% | 37.26% | plasma membrane |
| GH_D05G1005 | *GhNPF63* | 550 | 60446.04 | 9.23 | 48.36% | 11.45% | 4.18% | 36.00% | plasma membrane |
| GH_D05G1321 | *GhNPF64* | 579 | 65256.25 | 7.88 | 45.60% | 15.20% | 4.66% | 34.54% | plasma membrane |
| GH_D05G2519 | *GhNPF65* | 616 | 63840.83 | 8.53 | 44.32% | 14.77% | 3.57% | 37.34% | plasma membrane |
| GH_D05G2608 | *GhNPF66* | 617 | 68367.25 | 8.56 | 50.08% | 12.48% | 3.89% | 33.55% | plasma membrane |
| GH_D05G3384 | *GhNPF67* | 588 | 67711.08 | 8.26 | 38.44% | 11.56% | 2.38% | 38.44% | plasma membrane |
| GH_D06G0586 | *GhNPF68* | 586 | 65193.67 | 9.03 | 47.95% | 13.48% | 2.90% | 35.67% | plasma membrane |
| GH_D06G1229 | *GhNPF79* | 573 | 63536.85 | 6.61 | 49.21% | 14.66% | 4.36% | 31.76% | plasma membrane |
| GH_D06G1856 | *GhNPF70* | 597 | 63760.69 | 9.32 | 46.40% | 13.90% | 3.52% | 36.18% | plasma membrane |
| GH_D06G2094 | *GhNPF71* | 583 | 65938.48 | 7.9 | 49.23% | 14.75% | 3.60% | 32.42% | plasma membrane |
| GH_D07G0415 | *GhNPF72* | 581 | 67987.16 | 9.1 | 47.33% | 14.29% | 3.27% | 35.11% | plasma membrane |
| GH_D07G1248 | *GhNPF73* | 558 | 61254.14 | 8.87 | 46.77% | 12.90% | 4.12% | 36.20% | plasma membrane |
| GH_D07G1648 | *GhNPF74* | 604 | 66975.1 | 8.44 | 50.33% | 12.25% | 3.15% | 34.27% | plasma membrane |
| GH_D07G1855 | *GhNPF75* | 595 | 63559.64 | 9.21 | 49.08% | 13.45% | 4.03% | 33.45% | plasma membrane |
| GH_D07G2264 | *GhNPF76* | 609 | 65824.96 | 8.94 | 52.55% | 12.81% | 3.12% | 31.53% | plasma membrane |
| GH_D08G0062 | *GhNPF77* | 576 | 64964.63 | 7.52 | 46.18% | 12.67% | 4.17% | 36.98% | plasma membrane |
| GH_D08G1864 | *GhNPF78* | 599 | 64495.58 | 9.26 | 46.74% | 12.19% | 4.01% | 37.06% | plasma membrane |
| GH_D08G1974 | *GhNPF79* | 586 | 89983.17 | 8.94 | 47.44% | 11.77% | 3.75% | 37.03% | plasma membrane |
| GH_D08G2615 | *GhNPF80* | 587 | 66501.04 | 6.39 | 50.60% | 10.90% | 2.39% | 36.12% | plasma membrane |
| GH_D08G2830 | *GhNPF81* | 818 | 64255.17 | 8.45 | 54.52% | 13.57% | 3.18% | 28.73% | plasma membrane |
| GH_D09G1643 | *GhNPF82* | 594 | 66025.22 | 8.6 | 49.16% | 13.13% | 3.37% | 34.34% | plasma membrane |
| GH_D09G1746 | *GhNPF83* | 569 | 63797.02 | 8.49 | 46.40% | 14.24% | 3.51% | 35.85% | plasma membrane |
| GH_D10G0905 | *GhNPF84* | 596 | 65911.69 | 8.28 | 51.68% | 10.07% | 2.35% | 35.91% | plasma membrane |
| GH_D10G2083 | *GhNPF85* | 583 | 64980.4 | 8.91 | 48.20% | 13.55% | 3.26% | 34.99% | plasma membrane |
| GH_D10G2132 | *GhNPF86* | 584 | 64205.87 | 9.53 | 48.46% | 12.67% | 3.25% | 35.62% | plasma membrane |
| GH_D11G1638 | *GhNPF87* | 595 | 66063.53 | 9.08 | 51.60% | 13.61% | 3.03% | 31.76% | plasma membrane |
| GH_D12G0513 | *GhNPF88* | 576 | 64264.21 | 9.16 | 46.35% | 12.67% | 3.12% | 37.85% | plasma membrane |
| GH_D12G1864 | *GhNPF89* | 582 | 63722.2 | 6.79 | 51.20% | 11.17% | 2.41% | 35.22% | plasma membrane |
| GH_D12G1934 | *GhNPF90* | 602 | 66821 | 8.91 | 48.34% | 11.96% | 2.82% | 36.88% | plasma membrane |
| GH_D12G1937 | *GhNPF91* | 579 | 64008.95 | 9.03 | 49.22% | 12.09% | 3.28% | 35.41% | plasma membrane |
| GH_D13G0128 | *GhNPF92* | 616 | 63787.32 | 8.54 | 46.75% | 13.80% | 3.57% | 35.88% | plasma membrane |
| GH_D13G0710 | *GhNPF93* | 580 | 64025.97 | 8.81 | 50.34% | 10.86% | 3.28% | 35.52% | plasma membrane |
| GH_D13G1352 | *GhNPF94* | 562 | 61261.44 | 8.33 | 49.82% | 11.92% | 3.56% | 34.70% | plasma membrane |
| GH_D13G2098 | *GhNPF95* | 579 | 65364.7 | 5.86 | 48.88% | 13.64% | 2.59% | 34.89% | plasma membrane |
| GH_D13G2416 | *GhNPF96* | 598 | 68551.41 | 6.67 | 49.83% | 13.38% | 2.84% | 33.95% | plasma membrane |
| GH_A03G2453 | *GhNPF97* | 593 | 65762.28 | 7.19 | 47.89% | 10.96% | 2.19% | 38.95% | plasma membrane |
| GH_A12G2985 | *GhNPF98* | 586 | 64679.58 | 8.91 | 47.95% | 12.46% | 3.41% | 36.18% | plasma membrane |

Note: Ninety-eight *GhNPF*s were identified and their principle transcript ID, gene name, protein length (aa), molecular weight (Mw), isoelectric point (pI), secondary structure—alpha-helices (Hh), extended strands (Ee), beta turns (Tt) and random coils (Cc)—and localization were listed.

Table S3 Segmental duplication and tandem duplication gene pairs among *GhNPF* family members.

| **No.** | **Segmental duplication gene pairs** | **Tandem duplication gene pairs** |
| --- | --- | --- |
| 1 | *GhNPF1/47* | *GhNPF5/6* |
| 2 | *GhNPF2/49* | *GhNPF62/63* |
| 3 | *GhNPF3/15* |  |
| 4 | *GhNPF3/54* |  |
| 5 | *GhNPF3/64* |  |
| 6 | *GhNPF4/30* |  |
| 7 | *GhNPF4/57* |  |
| 8 | *GhNPF4/78* |  |
| 9 | *GhNPF5/31* |  |
| 10 | *GhNPF5/79* |  |
| 11 | *GhNPF7/29* |  |
| 12 | *GhNPF7/56* |  |
| 13 | *GhNPF7/61* |  |
| 14 | *GhNPF7/77* |  |
| 15 | *GhNPF8/55* |  |
| 16 | *GhNPF8/90* |  |
| 17 | *GhNPF9/51* |  |
| 18 | *GhNPF10/53* |  |
| 19 | *GhNPF11/18* |  |
| 20 | *GhNPF11/59* |  |
| 21 | *GhNPF11/67* |  |
| 22 | *GhNPF12/60* |  |
| 23 | *GhNPF13/29* |  |
| 24 | *GhNPF13/56* |  |
| 25 | *GhNPF13/61* |  |
| 26 | *GhNPF13/77* |  |
| 27 | *GhNPF14/62* |  |
| 28 | *GhNPF15/64* |  |
| 29 | *GhNPF16/65* |  |
| 30 | *GhNPF17/94* |  |
| 31 | *GhNPF18/59* |  |
| 32 | *GhNPF18/67* |  |
| 33 | *GhNPF19/38* |  |
| 34 | *GhNPF19/58* |  |
| 35 | *GhNPF19/86* |  |
| 36 | *GhNPF20/24* |  |
| 37 | *GhNPF20/68* |  |
| 38 | *GhNPF22/70* |  |
| 39 | *GhNPF22/92* |  |
| 40 | *GhNPF23/37* |  |
| 41 | *GhNPF23/71* |  |
| 42 | *GhNPF23/85* |  |
| 43 | *GhNPF24/72* |  |
| 44 | *GhNPF24/73* |  |
| 45 | *GhNPF26/28* |  |
| 46 | *GhNPF26/74* |  |
| 47 | *GhNPF26/76* |  |
| 48 | *GhNPF27/75* |  |
| 49 | *GhNPF28/76* |  |
| 50 | *GhNPF28/74* |  |
| 51 | *GhNPF29/56* |  |
| 52 | *GhNPF29/61* |  |
| 53 | *GhNPF29/77* |  |
| 54 | *GhNPF30/58* |  |
| 55 | *GhNPF30/78* |  |
| 56 | *GhNPF31/79* |  |
| 57 | *GhNPF32/81* |  |
| 58 | *GhNPF34/82* |  |
| 59 | *GhNPF35/83* |  |
| 60 | *GhNPF36/84* |  |
| 61 | *GhNPF37/71* |  |
| 62 | *GhNPF37/85* |  |
| 63 | *GhNPF38/59* |  |
| 64 | *GhNPF38/86* |  |
| 65 | *GhNPF39/75* |  |
| 66 | *GhNPF39/87* |  |
| 67 | *GhNPF41/91* |  |
| 68 | *GhNPF42/92* |  |
| 69 | *GhNPF43/93* |  |
| 70 | *GhNPF44/94* |  |
| 71 | *GhNPF45/95* |  |
| 72 | *GhNPF46/96* |  |
| 73 | *GhNPF51/89* |  |
| 74 | *GhNPF55/90* |  |
| 75 | *GhNPF56/61* |  |
| 76 | *GhNPF56/77* |  |
| 77 | *GhNPF57/78* |  |
| 78 | *GhNPF58/86* |  |
| 79 | *GhNPF59/67* |  |
| 80 | *GhNPF61/77* |  |
| 81 | *GhNPF75/87* |  |
| 82 | *GhNPF74/76* |  |
| 83 | *GhNPF70/92* |  |
| 84 | *GhNPF69/96* |  |

Table S4 The Ka and Ks values of homologous pairs.

| **Gene ID** | **Gene ID** | Ka | Ks | Ka/Ks |
| --- | --- | --- | --- | --- |
| GH_A01G1530 | GH_D01G1628 | 0.01 | 0.04 | 0.25 |
| GH_A02G0439 | GH_D02G0451 | 0.01 | 0.07 | 0.14 |
| GH_A02G1421 | GH_A05G1317 | 0.08 | 0.68 | 0.12 |
| GH_A02G1421 | GH_D03G0790 | 0.01 | 0.05 | 0.20 |
| GH_A02G1421 | GH_D05G1321 | 0.09 | 0.66 | 0.14 |
| GH_A03G0072 | GH_A08G1847 | 0.07 | 1.01 | 0.07 |
| GH_A03G0072 | GH_D03G1892 | 0.01 | 0.07 | 0.14 |
| GH_A03G0072 | GH_D08G1864 | 0.07 | 0.97 | 0.07 |
| GH_A03G0138 | GH_A08G1961 | 0.08 | 0.86 | 0.09 |
| GH_A03G0138 | GH_D08G1974 | 0.08 | 0.85 | 0.09 |
| GH_A03G0609 | GH_A08G0060 | 0.12 | 1.88 | 0.06 |
| GH_A03G0609 | GH_D03G1347 | 0.01 | 0.05 | 0.20 |
| GH_A03G0609 | GH_D05G0904 | 0.11 | 0.92 | 0.12 |
| GH_A03G0609 | GH_D08G0062 | 0.11 | 1.90 | 0.06 |
| GH_A03G0780 | GH_D03G1042 | 0.01 | 0.04 | 0.25 |
| GH_A03G0780 | GH_D12G1934 | 0.26 | 1.97 | 0.13 |
| GH_A03G1176 | GH_D02G1242 | 0.01 | 0.06 | 0.17 |
| GH_A03G1757 | GH_D02G1913 | 0.01 | 0.05 | 0.20 |
| GH_A04G1174 | GH_A05G3381 | 0.31 | 2.25 | 0.14 |
| GH_A04G1174 | GH_D04G1513 | 0.01 | 0.03 | 0.33 |
| GH_A04G1174 | GH_D05G3384 | 0.33 | 2.10 | 0.16 |
| GH_A04G1524 | GH_D04G1867 | 0.00 | 0.03 | 0.00 |
| GH_A05G0912 | GH_A08G0060 | 0.09 | 1.19 | 0.08 |
| GH_A05G0912 | GH_D03G1347 | 0.11 | 0.87 | 0.13 |
| GH_A05G0912 | GH_D05G0904 | 0.02 | 0.08 | 0.25 |
| GH_A05G0912 | GH_D08G0062 | 0.09 | 1.25 | 0.07 |
| GH_A05G1017 | GH_D05G1004 | 0.01 | 0.04 | 0.25 |
| GH_A05G1317 | GH_D05G1321 | 0.00 | 0.03 | 0.00 |
| GH_A05G2485 | GH_D05G2519 | 0.01 | 0.04 | 0.25 |
| GH_A05G2751 | GH_D13G1352 | 0.13 | 0.62 | 0.21 |
| GH_A05G3381 | GH_D04G1513 | 0.31 | 2.17 | 0.14 |
| GH_A05G3381 | GH_D05G3384 | 0.01 | 0.08 | 0.13 |
| GH_A05G4208 | GH_A10G2024 | 0.09 | 0.81 | 0.11 |
| GH_A05G4208 | GH_D04G0171 | 0.01 | 0.06 | 0.17 |
| GH_A05G4208 | GH_D10G2132 | 0.09 | 0.82 | 0.11 |
| GH_A06G0613 | GH_A07G0410 | 0.06 | 0.58 | 0.10 |
| GH_A06G0613 | GH_D06G0586 | 0.01 | 0.04 | 0.25 |
| GH_A06G1827 | GH_D06G1856 | 0.01 | 0.03 | 0.33 |
| GH_A06G1827 | GH_D13G0128 | 0.10 | 0.89 | 0.11 |
| GH_A06G2054 | GH_A10G1981 | 0.06 | 0.67 | 0.09 |
| GH_A06G2054 | GH_D06G2094 | 0.00 | 0.04 | 0.00 |
| GH_A06G2054 | GH_D10G2083 | 0.05 | 0.62 | 0.08 |
| GH_A07G0410 | GH_D07G0415 | 0.01 | 0.04 | 0.25 |
| GH_A07G1265 | GH_D07G1248 | 0.02 | 0.05 | 0.40 |
| GH_A07G1648 | GH_A07G2319 | 0.11 | 0.72 | 0.15 |
| GH_A07G1648 | GH_D07G1648 | 0.02 | 0.07 | 0.29 |
| GH_A07G1648 | GH_D07G2264 | 0.11 | 0.68 | 0.16 |
| GH_A07G1816 | GH_D07G1855 | 0.01 | 0.05 | 0.20 |
| GH_A07G2319 | GH_D07G2264 | 0.00 | 0.05 | 0.00 |
| GH_A07G2319 | GH_D07G1648 | 0.10 | 0.65 | 0.15 |
| GH_A08G0060 | GH_D03G1347 | 0.11 | 1.87 | 0.06 |
| GH_A08G0060 | GH_D05G0904 | 0.09 | 1.25 | 0.07 |
| GH_A08G0060 | GH_D08G0062 | 0.01 | 0.06 | 0.17 |
| GH_A08G1847 | GH_D03G1892 | 0.07 | 1.04 | 0.07 |
| GH_A08G1847 | GH_D08G1864 | 0.01 | 0.05 | 0.20 |
| GH_A08G1961 | GH_D08G1974 | 0.01 | 0.04 | 0.25 |
| GH_A08G2837 | GH_D08G2830 | 0.02 | 0.05 | 0.40 |
| GH_A09G1695 | GH_D09G1643 | 0.00 | 0.04 | 0.00 |
| GH_A09G1796 | GH_D09G1746 | 0.00 | 0.03 | 0.00 |
| GH_A10G1730 | GH_D10G0905 | 0.00 | 0.02 | 0.00 |
| GH_A10G1981 | GH_D06G2094 | 0.05 | 0.66 | 0.08 |
| GH_A10G1981 | GH_D10G2083 | 0.01 | 0.06 | 0.17 |
| GH_A10G2024 | GH_D04G0171 | 0.09 | 0.82 | 0.11 |
| GH_A10G2024 | GH_D10G2132 | 0.01 | 0.08 | 0.13 |
| GH_A11G1605 | GH_D07G1855 | 0.10 | 0.64 | 0.16 |
| GH_A11G1605 | GH_D11G1638 | 0.01 | 0.05 | 0.20 |
| GH_A12G1939 | GH_D12G1937 | 0.01 | 0.04 | 0.25 |
| GH_A13G0128 | GH_D13G0128 | 0.00 | 0.04 | 0.00 |
| GH_A13G0524 | GH_D13G0710 | 0.01 | 0.05 | 0.20 |
| GH_A13G1419 | GH_D13G1352 | 0.01 | 0.05 | 0.20 |
| GH_A13G2117 | GH_D13G2098 | 0.01 | 0.04 | 0.25 |
| GH_A13G2425 | GH_D13G2416 | 0.01 | 0.05 | 0.20 |
| GH_D02G1242 | GH_D12G1864 | 0.09 | 0.63 | 0.14 |
| GH_D03G1042 | GH_D12G1934 | 0.26 | 1.94 | 0.13 |
| GH_D03G1347 | GH_D05G0904 | 0.11 | 0.86 | 0.13 |
| GH_D03G1347 | GH_D08G0062 | 0.11 | 1.98 | 0.06 |
| GH_D03G1892 | GH_D08G1864 | 0.07 | 1.00 | 0.07 |
| GH_D04G0171 | GH_D10G2132 | 0.09 | 0.83 | 0.11 |
| GH_D04G1513 | GH_D05G3384 | 0.33 | 2.04 | 0.16 |
| GH_D05G0904 | GH_D08G0062 | 0.09 | 1.31 | 0.07 |
| GH_D06G1229 | GH_D13G2416 | 0.11 | 1.03 | 0.11 |
| GH_D06G1856 | GH_D13G0128 | 0.10 | 0.91 | 0.11 |
| GH_D07G1648 | GH_D07G2264 | 0.10 | 0.62 | 0.16 |
| GH_D07G1855 | GH_D11G1638 | 0.10 | 0.65 | 0.15 |

Note: non-synonymous substitutions (Ka) and synonymous substitutions (Ks).

## Supplementary Figures


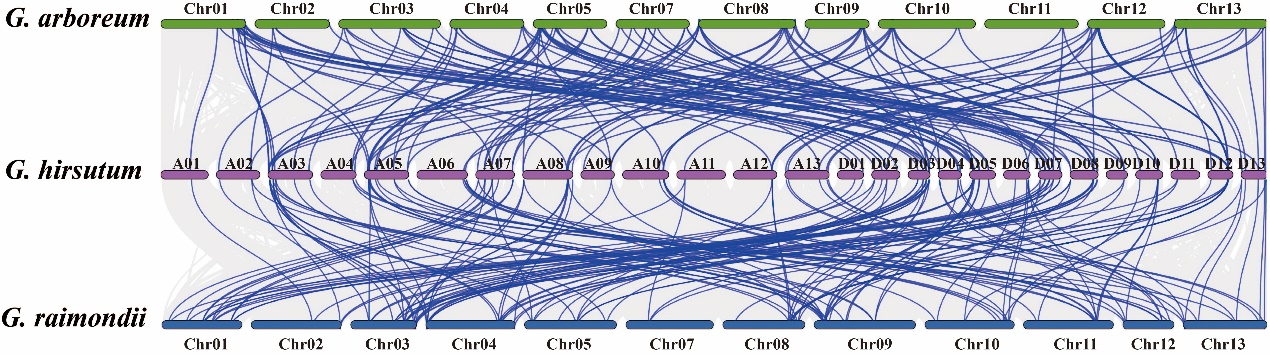


**Figure S1** Schematic diagram of the synteny assessment of *NPF* genes in cotton. The grey lines in the background revealed the collinear blocks while the blue lines highlight duplicated gene pairs.


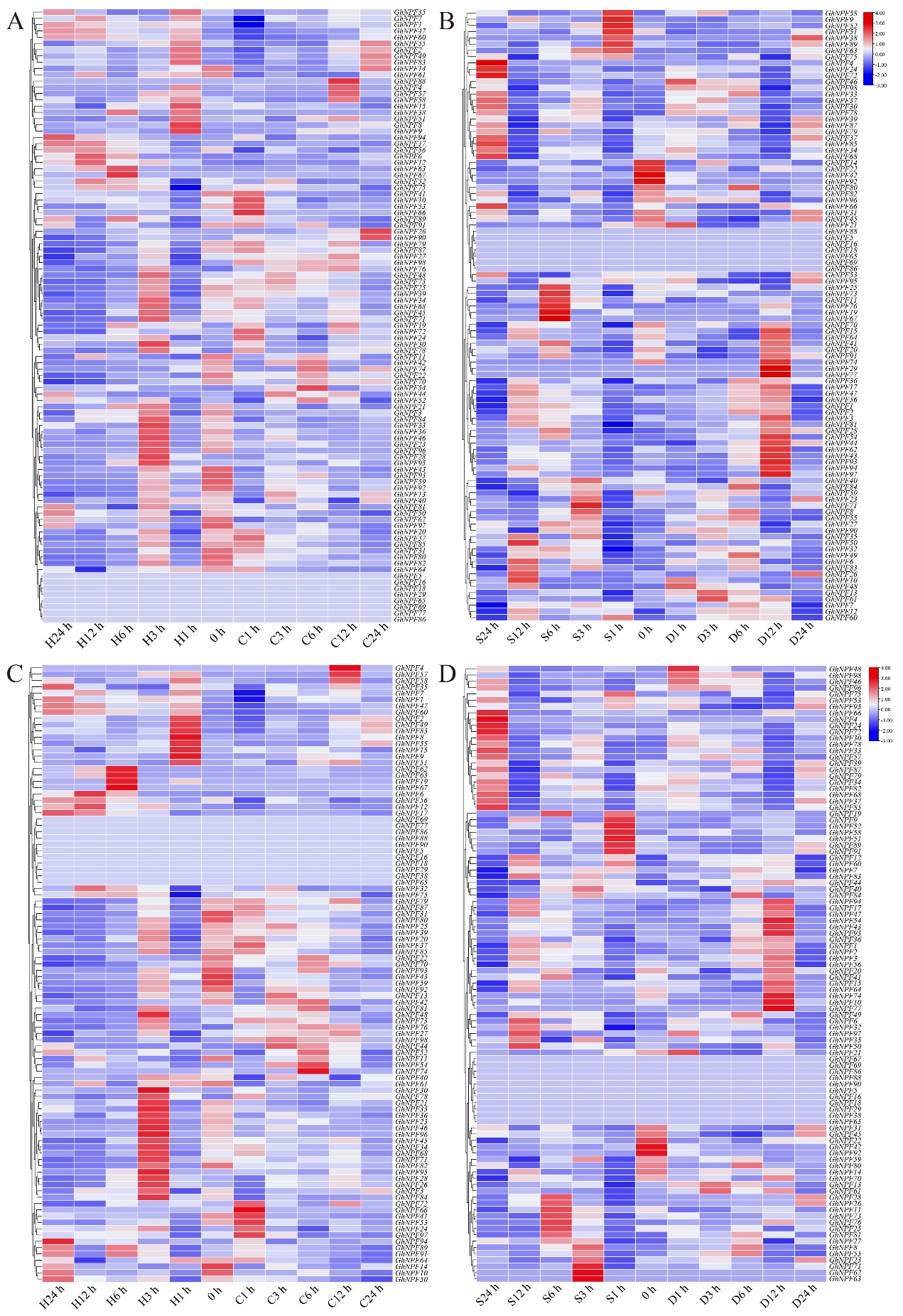


**Figure S2** Expression of 98 *GhNPF* genes in response to salt, peg and cold, heat treatments. (A, C) The expression patterns of 98 *GhNPFs* under cold and heat stresses were analyzed by RNA-seq at the ZJU and CRI. (B, D) The expression patterns of 98 *GhNPFs* under salt peg stresses were analyzed by RNA-sed at the ZJU and CRI.


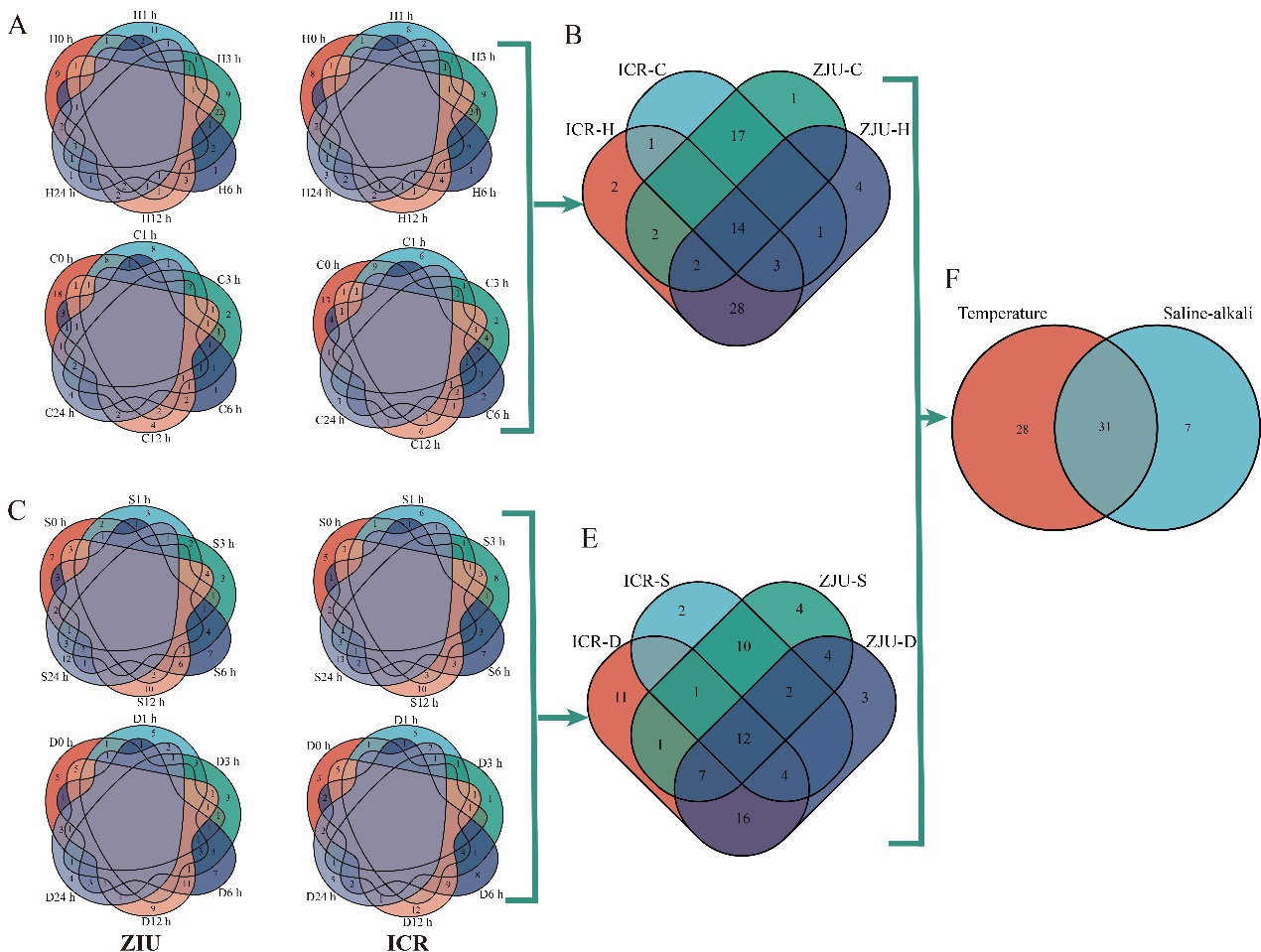


**Figure S3** Identification of the potential candidate genes associated with traits related to cotton resistance. (A, C) Distribution of highly expressed genes in different abiotic stress treatments at different times by the RNA-seq data from ZJU and ICR. (B, E) Venn diagram for the common genes with high expression levels between two RNA-seq data (ZJU and CRI). (F) Venn diagram of the common genes with high expression levels between temperature and saline-alkali treatment.
